# The Sweet Spot: Continued Search for the Glycemic Threshold for Macrovascular Disease—A Retrospective Single Center Experience

**DOI:** 10.5402/2012/874706

**Published:** 2012-10-14

**Authors:** Andrzej Boguszewski, Andrew Teklinski, Howard Rosman, Devang Desai, Sajid Ali, Susan Szpunar, Ruth Moore, James Maciejko

**Affiliations:** ^1^Department of Cardiology and Internal Medicine, St. John Hospital and Medical Center, Detroit, MI 48267-3271, USA; ^2^Michigan Heart & Vascular Institute, Petoskey, MI, USA; ^3^Sierra Nevada Cardiology Associates, Reno, NV, USA

## Abstract

*Background*. Atherosclerotic cardiovascular disease (ASCVD) is a common complication of diabetes mellitus and impaired fasting glucose (IFG). We hypothesized that the relation of fasting glucose levels to ASCVD is linear, with the prevalence of clinical ASCVD beginning to increase even among individuals currently categorized as normoglycemic. *Methods*. Patient charts were retrospectively reviewed from our Dyslipidemic Preventive Cardiology Clinic. We evaluated the prevalence of ASCVD relative to fasting glucose levels in a cross-section of patients at high risk for ASCVD. *Results*. In 558 dyslipidemic patients, ASCVD prevalence increased with increasing fasting glucose levels. A significantly higher prevalence of ASCVD was observed among patients with fasting glucose levels between 90 and 99 mg/dL versus lower levels. As glucose levels increased from 90 to 125 mg/dL, the prevalence of ASCVD continued to rise in parallel. Logistic regression analysis with forward likelihood ratio stepwise selection indicated that individuals with fasting blood glucose of 90–99 mg/dL were 2.6 times more likely to have ASCVD than those with lower levels of fasting blood glucose. *Conclusion*. Our findings suggest that the current cutoff for impaired fasting glucose of 100 mg/dL may be somewhat conservative and that a level above 90 mg/dL may be more appropriate as an ASCVD risk factor, particularly in patients with a lipid disorder.

## 1. Introduction

Atherosclerotic cardiovascular disease (ASCVD) is a common complication of diabetes mellitus (DM). Atherosclerosis is the primary reason for the decreased life expectancy of a newly diagnosed diabetic compared to an age and gender-matched nondiabetic [1, 2]. The risk of having a myocardial infarction over a period of seven years in the middle-aged diabetic patient without identified preexisting coronary heart disease (CHD) is the same as that in nondiabetic individual with existing CHD [3].

It is generally accepted that improved glycemic control decreases the onset and progression of microvascular complications, including nephropathy and retinopathy, and yet it does not reduce the risk of ASCVD and all-cause mortality [4, 5]. In the Prospective Pioglitazone Clinical Trial in macrovascular events (PROActive) study, however, improved glycemic control with pioglitazone reduced the composite endpoint of all-cause mortality, nonfatal myocardial infarction, and stroke in patients with type-2 diabetes and atherosclerosis [6]. In part, this may be due to the favorable effect of pioglitazone on the high-density lipoprotein (HDL), triglycerides, smaller LDL particles, and an improved low-density lipoprotein (LDL) to HDL cholesterol ratio in the pioglitazone group in the PROActive trial [6, 7].

The aim of our study was to determine the prevalence of clinical ASCVD among prediabetic patients and compare their characteristics to nondiabetic and diabetic patients. We hypothesized that clinical atherosclerotic disease is evident in the early glycemic spectrum of prediabetes.

## 2. Methods

We retrospectively reviewed 558 charts of patients referred over a four-year period to the Lipid Clinic at St. John Hospital and Medical Center. A history and physical examination, laboratory studies, and dietary evaluation were carried out during the patients first office encounter. Referring physicians also provided prior laboratory results and medical histories for these patients. The data analysis is limited to the 557 patients with complete information.

The patients were categorized, based upon highest fasting blood glucose value, in the categories <90 mg/dL, 90–99 mg/dL, 100–125 mg/dL, and 126+ mg/dL. The presence of diabetes mellitus (DM) was determined by patient history; the use of hypoglycemic agents (including insulin), or the presence of one or more fasting glucose levels ≥126 mg/dL, considered diagnostic of the disease [8, 9]. Patients previously diagnosed with DM were designated as diabetic regardless of fasting glucose levels.

For each glycemic category, the number of patients with clinically evident ASCVD was identified. ASCVD was defined as a history of coronary artery bypass graft surgery (CABG), percutaneous coronary intervention (PCI), myocardial infarction (MI), unstable angina, cerebrovascular accident (CVA), transient ischemic attack (TIA), or peripheral intervention [10]. 

Risk factors for ASCVD were also identified and included body mass index (BMI), tobacco use history (never, past use if quit >6 months previously, or active), age, gender, lipid phenotype (Table 1), family history of ASCVD, and presence of hypertension (HTN). Lipid phenotypes were determined based on the guidelines for LDL-C and fasting triglycerides of the National Cholesterol Education Program Adult Treatment Panel III report as shown in [11] and the Fredrickson classification [12]. Patients were excluded if they were under 18 years of age, if any of these variables were not documented, or if there were no fasting glucose values available.

Data were analyzed using chi-squared tests for categorical variables and ANOVA for continuous variables. The crude association between glycemic category and presence of ASCVD was also examined using the chi-squared test for trend. For ANOVA, when the overall *F* test was significant, multiple pairwise comparisons using the Bonferroni correction of the *P* value were conducted. Stepwise forward logistic regression was used to predict the probability of ASCVD based upon significant predictors identified in univariate analysis and fasting glucose status. All analyses were completed with SPSS v. 19.0, and a *P* value of 0.05 or less was considered to indicate statistical significance. This project was approved by the St. John Institutional Review Board.

## 3. Results

Baseline clinical demographics are provided in Table 1. The mean age was 54.9 ± 13.2 (SD) years, and 56% of the subjects were male. The average body mass index was 30.0 ± 5.8 kg/m^2^. Using the NIH classifications for BMI, 39.3% (219) of the study group were overweight, and 44% (245) were obese [13]. Half the patients had never smoked while 31% were current smokers. For glycemic category, 21.7% (121/557) were in the <90 mg/dL category, 25.5% (142) in the 90–99 mg/dL category, 34.6% (193/557) in the 100–125 mg/dL category and 17.9% (100/557) in the 126 mg/dL and greater category. Fifty-five percent were being treated for HTN, and 39% had clinically evident ASCVD.

Table 2 displays the association between various demographic and clinical factors by glycemic classification. ANOVA indicated a significant difference in mean age by glycemic classification (*P* < 0.0001). From pairwise comparisons, the mean age for the <90 mg/dL group was significantly lower than the 100–125 and 126+ mg/dL groups (*P* < 0.0001) and the mean age for the 90–99 mg/dL group was significantly lower than the 100–125 and 126+ mg/dL groups (both, *P* = 0.045). As expected, ANOVA also indicated a significant difference in mean BMI among the groups (*P* < 0.0001). From pairwise comparisons, the mean BMI for the <90 mg/dL group was lower than the 100–125 mg/dL group (*P* = 0.003), and the mean BMI for the 126+ mg/dL group was significantly higher than all other groups (*P* < 0.0001, all comparisons). Women were less likely to be in the higher glycemic categories than men (*P* = 0.03). Smoking status, family history of CVD, HTN, and ASCVD also differed by glycemic classification (*P* < 0.0001). 

**Figure 1:**
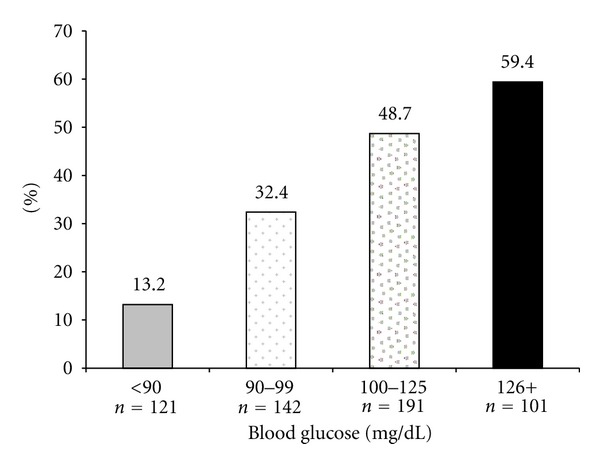
Prevalence of ASCVD by glucose category.

Figure 1 displays the unadjusted prevalence of ASCVD by glycemic classification. ASCVD prevalence differed across these groups (chi square, *P* < 0.0001), showing a linear trend of increase (chi-square test for trend, *P* < 0.0001).

Table 3 shows the association between the various clinical and demographic factors and the presence of ASCVD. The prevalence of ASCVD was higher in men (0.04), current and former smokers (*P* < 0.0001), subjects with hypertension (*P* < 0.0001), subjects with higher fasting blood glucose (as noted above), and subjects with higher BMI (*P* = 0.03). Subjects with ASCVD were also significantly older that subjects with no evidence of ASCVD (*P* < 0.0001).

Multiple logistic regression using a forward stepwise algorithm was used to predict the probability of having ASCVD given the independent predictors that were found to be related to ASCVD (as seen in Table 3). As seen in Table 4, after controlling for age, gender, BMI, hypertension, and smoking, the probability of having ASCVD increased 2.6 times in the glycemic category 90–99 mg/dL compared to <90 mg/dL, 3.4 times in the glycemic category 100–125 mg/dL compared to <90 mg/dL, and 4.1 times in the glycemic category 126+ mg/dL compared to <90 mg/dL. BMI dropped out of the model.

## 4. Discussion

The core metabolic defect contributing to the development of type 2 DM is insulin resistance, detected in more than 92% of diabetics [3]. Insulin resistance is the primary and earliest pathogenic defect to appear. Even in the absence of overt hyperglycemia, insulin resistance is associated with a cluster of abnormalities that increase the risk of ASCVD, including dyslipidemia, activation of procoagulants, and endothelial cell dysfunction [14, 15]. Impaired pancreatic beta-cell function becomes an increasingly important feature as type 2 DM progresses, altering the insulin responses to both glucose and amino acids. As the beta cell continues to fail, relative insulin deficiency coupled with impaired glucose tolerance triggers overt type 2 diabetes mellitus [16]. 

The role of insulin resistance in the sequential development of type 2 diabetes mellitus highlights the importance of recognizing the impaired fasting glucose (100–125 mg/dL) to identify individuals with insulin resistance at an earlier stage. Insulin resistance and its association with increased risk of ASCVD can be present with normal fasting glucose levels (<100 mg/dL) [14]. Any deviation of glucose above the normal range levels has been associated with an increased relative risk for myocardial infarction and stroke [17, 18]. However, a glycemic threshold for macrovascular-associated mortality or onset of disease has been elusive [19]. 

In this cohort, those with fasting glucose values <100 mg/dL are likely to be a mixed population, composed of individuals with normal and impaired glucose homeostasis. The increased prevalence of ASCVD in this sample makes it difficult to extrapolate risk based on fasting glucose to another individual. Despite this limitation, our data suggests that, in this population of hyperlipidemic patients, ASCVD prevalence relative to fasting glucose levels is a continuum. ASCVD increases as glucose levels rise (Figure 1), with a stair-step when glucose levels exceed 89 mg/dL, and a continued rise in each IFG subgroup. 

The literature is mixed on whether macrovascular disease is seen at lower fasting glucose levels, not providing enough evidence to change primary prevention management [17–21]. Our retrospective study is only hypothesis generating. Plus, if true, it might signify only that dyslipidemic patients are more susceptible to elevations of glucose. Whether the glycemic threshold for development of macrovascular disease in this population is truly 90 (mg/dL) will require further study.

The limitations of this study include its retrospective design and the population studied; namely, patients referred with difficulty to treat dyslipidemia. Our findings must be considered as only hypothesis generated. Also our hypothesis may not be valid for individuals with lipid values controlled in the normal range with standard therapy. Prospective clinical trials are needed to better classify morbidity and mortality in regards to glucose levels and the risk of ACVD in patients with and without dyslipidemia.

## Figures and Tables

**Table 1:** Demographic and clinical characteristics of the study population (*n* = 558).

Variable	Mean ± SD or percent (*N*)
Age (yrs; mean ± SD)	54.9 ± 13.2
BMI (kg/m^2^)	30.0 ± 5.8
Male gender	56% (312)
Nonsmoker	50% (274)
Current smoker	31% (173)
Past smoker (quit > 6 mths.)	19% (107)
Hypertension	55% (304)
Positive family history (*n* = 422)	40% (167)
Hypercholesterolemia (type IIa)	64% (355)
Mixed hyperlipidemia (type IIb)	28% (155)
Isolated hypertriglyceridemia (type IV)	8% (46)
Normoglycemic	42.5% (237)
Impaired glucose tolerance	28.9% (161)
Diabetic	28.7% (160)
ASCVD	216 (39%)

ASCVD: atherosclerotic cardiovascular disease.

**Table 2:** Associations between glycemic classification and various demographic and clinical variables.

	Fasting blood glucose category				
Characteristic	<90 mg/dL	90–99 mg/dL	100–125 mg/dL	126+ mg/dL	*P* value
	(*n* = 121)	(*n* = 142)	(*n* = 193)	(*n* = 100)	
Age (mean ± s.d.)	49.8 ± 14.7^†^	53.6 ± 13.9*	57.4 ± 11.4	58.1 ± 11.4	<0.0001
BMI	28.0 ± 5.1^§^	28.8 ± 5.6	30.3 ± 5.3	33.5 ± 6.1^‡^	<0.0001
% Male	47.9%	55.6%	55.4%	67.3%	0.04
Smoking					
Never	70.0%	56.0%	41.9%	30.7%	<0.0001
Current	10.8%	26.2%	40.3%	44.6%	
Past	19.2%	17.7%	17.8%	24.8%	
Positive family history of CVD	48.8%	52.6%	24.1%	50.7%	<0.0001
Presence of HTN	38.3%	41.8%	65.6%	71.3%	<0.0001
ASCVD	13.2%	32.4%	48.7%	59.4%	<0.0001^*ℓ*^

^†^On multiple pairwise comparisons (Bonferroni correction of *P* value) the mean age for the patients with fasting blood glucose <90 was significantly different from the 100–125 and 126+ groups, respectively (*P* < 0.0001).

*On multiple pairwise comparisons (Bonferroni correction of *P* value) the mean age for the patients with fasting blood glucose 90–99 was significantly different from the 100–125 and 126+ groups, respectively (*P* = 0.045).

^§^On multiple pairwise comparisons (Bonferroni correction of *P* value) the mean BMI for the patients with fasting blood glucose <90 was significantly different than the 100–125 group (*P* = 0.003).

^‡^On multiple pairwise comparisons (Bonferroni correction of *P* value) the mean BMI for the patients with fasting blood glucose 126+ was significantly different from all of the other groups (*P* < 0.0001).

^*ℓ*^Both the Pearson chi-square and the chi-square test for trend were statistically significant (*P* < 0.0001).

**Table 3:** Associations between ASCVD and various demographic and clinical variables.

Characteristic	Presence of ASCVD		*P* value
	Yes	No
Age (mean ± s.d.)	60.1 ± 10.7	51.6 ± 13.6	<0.0001
Gender			
Male	42.6%	57.4%	0.04
Female	34.1%	65.9%	
Family history of CVD			
Yes	34.7	65.3	NS
No	40.9	59.1	
Smoking			
Never	25.5	74.5	<0.0001
Current	56.4	43.6	
Past	45.3	54.7	
HTN			
Yes	52.1	47.9	<0.0001
No	23.2	76.8	
BMI category			
<18.5 (underweight)	0.0%	100%	0.03
18.5–24.9 (normal)	30%	70%	
25.0–29.9 (overweight)	36.7%	63.3%	
30+ (obese)	44.7%	55.3%	

The association of ASCVD with glucose category is shown in Table 2 and Figure 1 and not repeated here.

**Table 4:** Results of logistic regression predicting the probability of ASCVD.

Characteristic	Odds ratio	*P* value	95% confidence interval
Age	1.05	<0.0001	1.03, 1.07
Female gender	0.55	0.005	0.36, 0.83
HTN	2.59	<0.0001	1.68, 3.96
Past or current smoker	2.59	<0.0001	1.72, 3.91
Glucose 90–99*	2.55	0.008	1.28, 5.06
Glucose 100–125*	3.38	<0.0001	1.76, 6.48
Glucose 126+*	4.10	<0.0001	1.96, 8.41

*Compared to baseline category of glucose <90 mg/dL.
